# Robust detection of undifferentiated iPSC among differentiated cells

**DOI:** 10.1038/s41598-020-66845-6

**Published:** 2020-06-24

**Authors:** Keisuke Sekine, Syusaku Tsuzuki, Ryota Yasui, Tatsuya Kobayashi, Kazuki Ikeda, Yuki Hamada, Eriko Kanai, J. Gray Camp, Barbara Treutlein, Yasuharu Ueno, Satoshi Okamoto, Hideki Taniguchi

**Affiliations:** 1 Division of Regenerative Medicine, Center for Stem Cell Biology and Regenerative Medicine, The Institute of Medical Science, The University of Tokyo, Tokyo, Japan; 2 Department of Regenerative Medicine, Yokohama City University Graduate School of Medicine, Yokohama, Japan; 3 Eiken Chemical Co., Ltd., Tokyo, Japan; 4 Institute of Molecular and Clinical Ophthalmology Basel, Basel, Switzerland; 5 Quantitative Developmental Biology Lab, ETH Zurich, Zurich, Switzerland; 6 Advanced Medical Research Center, Yokohama City University, Yokohama, Japan

**Keywords:** Stem-cell biotechnology, Stem-cell biotechnology, Induced pluripotent stem cells, Induced pluripotent stem cells

## Abstract

Recent progress in human induced pluripotent stem cells (iPSC) technologies suggest that iPSC application in regenerative medicine is a closer reality. Numerous challenges prevent iPSC application in the development of numerous tissues and for the treatment of various diseases. A key concern in therapeutic applications is the safety of the cell products to be transplanted into patients. Here, we present novel method for detecting residual undifferentiated iPSCs amongst directed differentiated cells of all three germ lineages. Marker genes, which are expressed specifically and highly in undifferentiated iPSC, were selected from single cell RNA sequence data to perform robust and sensitive detection of residual undifferentiated cells in differentiated cell products. *ESRG (Embryonic Stem Cell Related)*, *CNMD (Chondromodulin)*, and *SFRP2 (Secreted Frizzled Related Protein 2)* were well-correlated with the actual amounts of residual undifferentiated cells and could be used to detect residual cells in a highly sensitive manner using qPCR. In addition, such markers could be used to detect residual undifferentiated cells from various differentiated cells, including hepatic cells and pancreatic cells for the endodermal lineage, endothelial cells and mesenchymal cells for the mesodermal lineage, and neural cells for the ectodermal lineage. Our method facilitates robust validation and could enhance the safety of the cell products through the exclusion of undifferentiated iPSC.

## Introduction

Induced pluripotent stem cell (iPSC) technologies could pave the way for patients to reap the benefits of regenerative medicine and therapies^1–3^. Theoretically, iPSC have the potential to differentiate into any type of cells in our body, and protocols have been developed to differentiate iPSCs into specific cell types, including neural cells, cardiomyocytes, chondrocytes, retinal pigment epithelial cells, pancreatic islet cells, and hepatocytes^4^. Several approaches, in addition to standard 2D culture, have been explored for direct cell differentiation. Organoid technology has emerged as a tool for mimicking miniaturized organs from stem cells, and culturing them into buds using iPSC technologies^5–12^. We also previously developed a method for the generating multicellular 3D miniaturized liver primordia organoids from pluripotent stem cells^13–15^.

To apply such iPSC technologies to regenerative medicine and offer their potential benefits to patients, researchers are working to ensure the functionality and safety of iPSC-derived cells following transplantation. For example, the safety of iPSC has been enhanced through various approaches, such as the application of L-Myc as opposed to c-Myc proteins, use of non genome-integrative methods, and the validation of undifferentiated cells^16,17^. Nevertheless, it is essential to ensure that the undifferentiated cells are excluded from differentiated cells, which could be used in transplantation activities, since cells exhibiting pluripotency have the capacity to generate teratomas^18–20^.

Several reports have explored strategies of excluding and/or detecting undifferentiated cells in differentiated cells^21–23^. One method cultures undifferentiated cells among differentiated cells while maintaining an iPSC state, and could detect undifferentiated cells in differentiated cells efficiently^22^. In addition, growth medium could be modified to exclude residual undifferentiated cells from cardiomyocytes^24^. Cell sorting with cell surface markers has been reported to purify intermediate cell lineages used to derive dopaminergic neurons^25^, while *LIN28A (Lin-28 Homolog A)* could be used to detect residual undifferentiated cells in iPSC-derived differentiated retinal pigment epithelial (RPE) cells^21^, which was already applied to patients. Such methods are often optimized for specific differentiation protocols and are not always applicable to the other lineages. Therefore, it is critical to develop more versatile methods to facilitate the detection of residual undifferentiated cells in differentiated cells. Here, we report a method for detecting undifferentiated cells amongst iPSC-derived cells in all three germ layers.

## Results

### *Lin28A* is not suitable for detecting undifferentiated iPSC in hepatic differentiation

**Figure 1:**
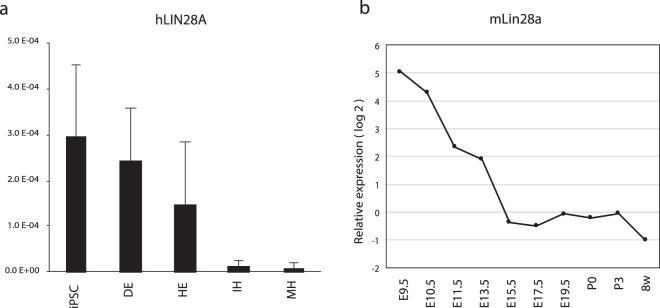
*LIN28A* is not suitable for detecting undifferentiated iPSC during hepatic differentiation. (**a**) Human *LIN28A* expression during hepatic differentiation from iPSC. DE, definitive endoderm; HE, hepatic endoderm; IH, immature hepatocyte; MH, mature hepatocyte. The relative expression levels were normalized by the amount of 18S rRNA in each sample. (**b**) Mouse *Lin28a* expression in hepatic cells during liver development. For samples from embryonic day 9.5 (E9.5) to post natal day 3 (P3) total RNA was isolated from nonhematopoietic (CD45 − TER119−) cells. For 8 week (8w) old sample, hepatic cell fraction was isolated by centrifugation.

*LIN28A*, formerly referred to as *LIN28*, has been reported previously to be a marker for residual undifferentiated iPSC in iPSC-derived RPE^3^. *LIN28A* expression was examined to validate the potential application of *LIN28A* in the detection of residual undifferentiated cells during iPSC differentiation toward hepatic lineage cells. While *LIN28A* expression was high in hepatic endoderm (HE), it remained unaltered in the immature hepatocyte (IH) stage (Figs. 1a and S1). We considered two possible explanations for the observation. One is that *LIN28A* is expressed in hepatic lineage cells and; therefore, is not suitable for the detection of undifferentiated iPSC in hepatic lineage cells. The other potential explanation is *LIN28A* is actually the undifferentiated iPSC marker and there were undifferentiated iPSCs in the differentiated cells in the present study. To explore the possibility of the above cases, we evaluated gene expression in the developing mouse liver and observed that hepatic cells expressed some amounts of mouse *Lin28a* during liver development (Fig. 1b). This result suggests that *Lin28a* express during hepatocyte differentiation and might not suitable to detect undifferentiated cells in differentiated, but immature hepatic progenitors.

Subsequently, we evaluated whether undifferentiated iPSCs were present in the differentiated cells in the present study. We utilized “re-seeding method”, by which we re-seeded differentiated cells and cultivated them for ~1 week in iPSC maintenance state to derive undifferentiated cell colonies to facilitate the direct observation of the contamination with undifferentiated cells in the culture^22^.

To validate this re-seeding method, we spiked-in (mixed) undifferentiated iPSC to the differentiated cells and detected at least 0.0025% of spiked-in undifferentiated cells in our condition (data not shown). Notably, the method is robust and the more cells are seeded in culture, the more the detection limit can be lowered, although it requires at least 1 week to grow undifferentiated cell colonies. No undifferentiated cell colonies were detected from HE cells when cells were seeded at densities of 8 × 10^4^ cells/cm^2^ and 1.6 × 10^5^ cells in three independent experiments. The results indicate that *LIN28A* is not suitable for detecting undifferentiated iPSC in hepatic differentiation (Fig. 1 and see below).

### Identification of a marker gene for residual undifferentiated iPSC

We have previously reported the use of single-cell RNA sequencing (scRNAseq) for the reconstruction of hepatocyte-like lineage development from pluripotency under two-dimensional culture^14^. We explored our scRNAseq data, and we selected genes consistent with following criteria: (1) Specific expression in the iPSC stage to exclude genes expressed during directed hepatic differentiation, (2) high expression in iPSC to facilitate high-level and sensitive detection even at low levels of undifferentiated iPSC contamination, and (3) considerable difference in expression level between iPSC and target cells i.e., hepatic endoderm (HE) cells.

**Figure 2:**
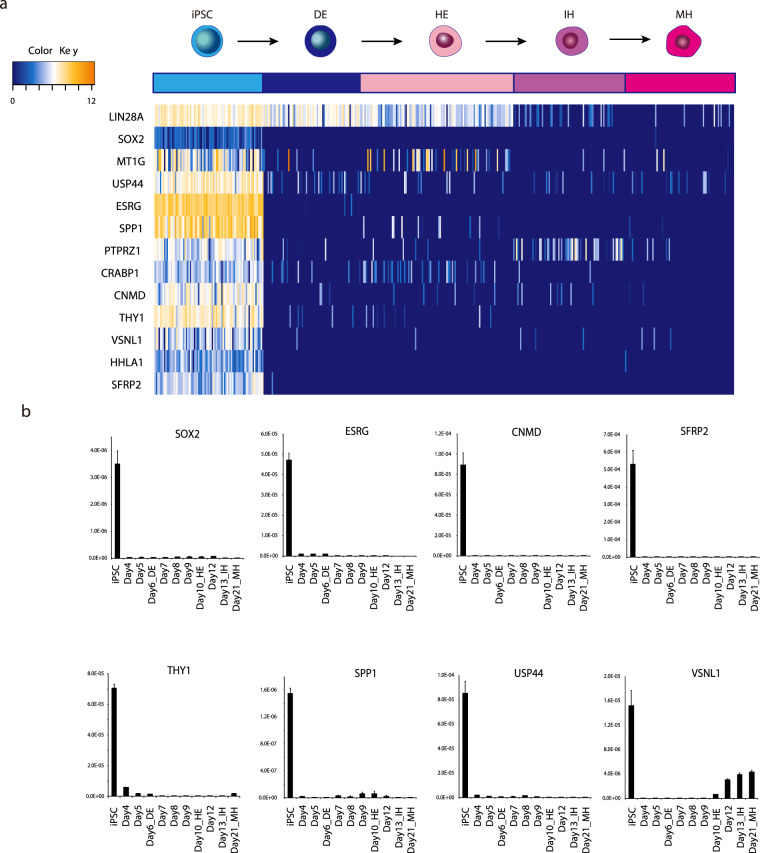
Identification of candidate genes for detecting undifferentiated iPSC during hepatic differentiation. (**a**) Single-cell RNA sequence analysis identified novel candidate genes. Upper illustration and color column represents differentiation lineages from undifferentiated iPSC to mature hepatocyte (MH). DE: definitive endoderm, HE: hepatic endoderm, IH: immature hepatocyte. (**b**) qPCR analysis of marker gene candidates during hepatic differentiation. Down regulation of gene expressions during hepatic differentiation. The relative expression levels were normalized by the amount of 18S rRNA in each sample.

Twelve genes were selected as illustrated in Fig. 2a which expressed highly, specifically, and abundantly in iPSC. Marker gene expression was confirmed using quantitative reverse transcription-polymerase chain reaction (qPCR), and *ESRG (Embryonic Stem Cell Related)*, *SFRP2 (Secreted Frizzled Related Protein 2)*, *CNMD(Chondromodulin, also referred to as LECT1)*, *SOX2 (SRY-Box 2)*, *THY1 (Thy-1 Cell Surface Antigen)*, *USP44 (Ubiquitin Specific Peptidase 44)*, *VSNL1 (Visinin Like 1)*, and *SPP1 (Secreted Phosphoprotein 1)* were selected for further analyses (Fig. 2b). Marker genes were also checked using several iPSC lines to exclude clone specific variations. We confirmed that the genes were down-regulated considerably following the stimulation of differentiation in several other iPSC lines (Fig. S2). We estimated the actual RNA count of these markers in one undifferentiated iPSC by droplet digital PCR (Fig. S3). These results show that not only the expression level in undifferentiated iPSC is critical but also the downregulation in differentiated cell is important.

The results indicate that the genes fit the criteria described above as candidate marker genes for detecting residual undifferentiated cells in differentiated cells.

### Correlation of marker gene expression with actual residual undifferentiated cells and detection using single-molecule FISH

We explored whether expression levels of the candidate genes are correlated to residual undifferentiated iPSCs. To obtain actual residual undifferentiated iPSCs rather than spiked-in undifferentiated iPSC, we evaluated various iPSC derived cells and found that if we used iPSC which passage number was over 35, doubling time become faster than 20.5 hr, which turn out to related to increase the residual undifferentiated iPSC number. We used this over passaged cells as a model of actual residual undifferentiated iPSCs after differentiation (Fig. S4).

**Figure 3:**
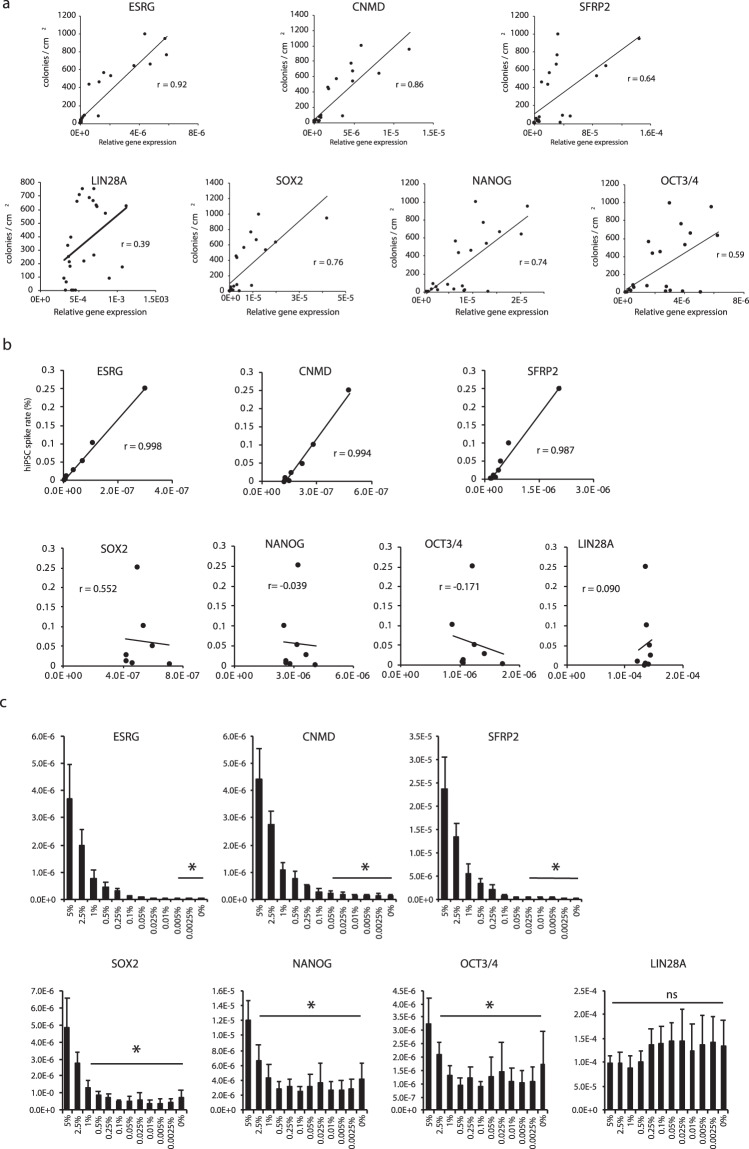
Correlation of marker gene expression with practical residual undifferentiated cells. (**a**) Correlation between gene expression and residual undifferentiated iPSC in HE. X–axes represent relative gene expression by qPCR and Y–axes represent actual residual colony number per cm^2^ when HE cells were re-seeded at a density of 8 × 10^4^ cells/cm^2^. (**b**) Correlation between spiked-in iPSC and gene expression of novel marker genes. X–axes represent relative gene expression by qPCR and Y–axes represent spiked-in undifferentiated iPSC percentage. (**c**) Detection limits of residual undifferentiated iPSC for novel markers and well-known iPSC marker genes. *p values <0.05. The all relative expression levels were normalized by the amount of 18S rRNA in each sample.

Various samples were evaluated for gene expression and simultaneously cultured in iPSC maintenance state. *ESRG*, *CNMD*, *SFRP2*, *SOX2*, and *NANOG* were well-correlated^26,27^ (r ≥ 0.6) with the numbers of residual undifferentiated iPSCs, whereas *OCT4* and *LIN28A* were not well-correlated with residual iPSC (Fig. 3a). Spike-in experiments were performed to determine the correlation between low number of spiked-in iPSC and gene expression. *ESRG*, *CNMD*, *SFRP2* were well-correlated (r ≥ 0.9) with the number of spike-in undifferentiated iPSCs, whereas *SOX2*, *OCT4* and *LIN28A* were not well-correlated with the number of spiked-in iPSC (Fig. 3b).

Detection limits were calculated by evaluating whether some spiked-in cells could be distinguished from non spiked-in cells the HE sample. Detection limits for *ESRG*, *CNMD* and *SFRP2* were 0.005%, 0.025%, and 0.025%, respectively (Fig. 3c), while the detection limits for *SOX2*, *NANOG*, and *OCT4* were 1%, 5%, and 2.5%, respectively. *LIN28A* could not discriminate 5% of undifferentiated iPSC in the spiked-in sample from HE cells. Therefore, *ESRG* and *CNMD* were considered suitable for sensitive detection of residual undifferentiated cells.

### *ESRG* and *CNMD* are robust residual undifferentiated iPSC markers for lineages derived from three germ layers

**Figure 4:**
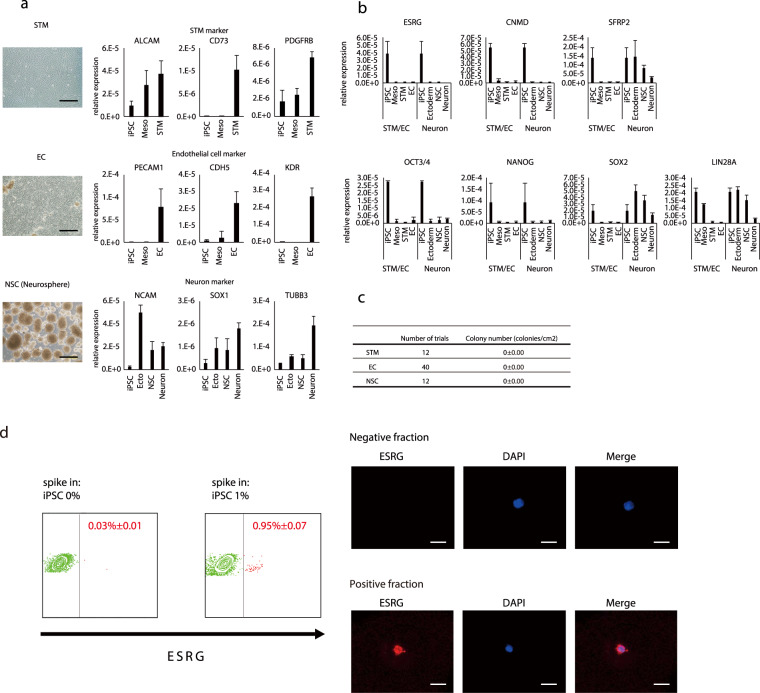
Novel markers were suitable for detecting residual cells from three germ lineages. (**a**) Mesoderm and ectoderm lineage cells were successfully differentiated based on gene expressions. Representative image of differentiated cells and marker gene expressions of each lineage. bar: 500 um. (**b**) Marker genes were down-regulated in the course of differentiation in each lineage and (**c**) residual iPSC in each lineage. The relative expression levels were normalized by the amount of 18S rRNA in each sample. (**d**) smFISH detection of undifferentiated cells by *ESRG* gene expression with flow cytometry. Positive fraction specifically included stained, *ESRG*-positive cell. bar: 10 um.

We wondered whether the marker genes could be applied in the detection of residual undifferentiated iPSC in iPSC derived lineage from other germ layers. Hepatic cells belong to the endodermal cell lineage. We also examined pancreatic cells, as endodermal lineage cells. Marker gene expression was downregulated markedly following differentiation and no residual undifferentiated cells were detected by cultivation (data not shown). Subsequently, we evaluated iPSC derived septum transversum mesenchyme (STM)/mesenchymal cells (MC) and endothelial cells (EC) as mesoderm derived lineages^15^. The marker genes were down-regulated in STM/MC and EC (Fig. 4a). Residual undifferentiated iPSC were not detected by the cultivation method. The results suggest that the markers were also useful for mesoderm evaluation.

Neuronal cell lineage was also evaluated as an ectoderm derived lineage. The marker genes, *ESRG* and *CNMD*, but not *SFRP2*, were down regulated in neural crest cells and neural stem cells (Fig. 4a–c), while *LIN28A* was not down-regulated during differentiation within the culture period. Residual undifferentiated iPSC were not detected using the cultivation method. The results suggest that the marker genes were also suitable for ectoderm derived lineages. To consolidate the above observations, we employed another method to derive three germ layer derived cells using a commercially available differentiation kit (Fig. S5). Three germ lineage cells were derived and the qPCR analysis and the cultivation evaluation were performed. According to the results, *ESRG* and *CNMD* are highly sensitive and robust markers for detecting residual undifferentiated cells in human iPSC-derived cell products differentiated into each of the three germ cell lineages. We also explored another method for detecting *ESRG* expression to expand the range of markers that could be applied. We applied the single-molecule FISH (smFISH) method followed by flow cytometry analysis. According to the results, smFISH was also applicable for the detection of *ESRG *+ cells in the differentiated cells (Fig. 4d).

## Discussion

The application of human iPSC in regenerative medicine could benefit patients suffering from various diseases greatly. Researchers and doctors are attempting to deliver novel therapies as rapidly as possible, while carefully assessing the safety of such novel therapies. Here, we describe a novel method for validating residual undifferentiated iPSC in differentiated cells. We evaluated *LIN28A* as a marker for residual undifferentiated cells and found it unsuitable for detecting residual cells in hepatocytic differentiation, in addition to in the early stages of neuronal differentiation.

We identified several marker genes suitable for hepatic differentiation, including *ESRG*, *CNMD*, and *SFRP2*. These marker genes satisfied several requirements as marker for residual undifferentiated iPSC. These genes highly and specifically expressed in the iPSC stage. They down-regulate immediately after differentiation and considerable difference in expression level between iPSC and target cells i.e., HE. These characteristics enables high-level and sensitive detection even at low levels of undifferentiated iPSC contamination, In addition, *ESRG* and *CNMD* were suitable for other cells derived from other germ layers. Therefore, the method is rapid and robust for the detection of residual undifferentiated iPSC in cells derived from three germ layers. The markers identified in the present study include well-known iPSC genes such as *ESRG*; however, not all “iPSC genes” are suitable for detecting residual undifferentiated cells. In fact, although *SOX2* or *NANOG* are the well-known iPSC genes that are expressed exclusively in iPSC, detection limits of the two genes for residual iPSC were relatively high compared to the genes identified in the present study. *LIN28A* have been reported to be a residual cell marker, however we observed low relation to the gene expression and the actual residual cell number. Several other methods for detection and elimination of undifferentiated iPSC were also reported including utilizing *microRNA-302*, cytotoxic viral vectors, rBC2LCN lectin, drug, antibody, or methionine removal. The detection limits of these method might be differ between target products and also, adaptation of these method to the on going research may require additional evaluation of product and optimization of established differentiation protocol^23,24,28–30^. Nevertheless, it is important to validate whether the detection method employed is suitable for their cell products.

Currently, numerous studies are exploring the potential application of iPSC technologies in in regenerative medicine, so that actual application could be witnessed in the near future. Although there are no full-proof strategies for ensuring the safety of technologies, it remains critical to evaluate technologies and products adopted in cell therapies. There are at least two bottlenecks in any method used to detect contamination of undifferentiated iPSC when large numbers of cells are being evaluated. If the large numbers of cells were analyzed over detection limits, the signal of residual iPSC would be suppressed by the signals from other differentiated cells. In addition, there are limitations with regard to the amount of products that could be input in a validation assay, such as the amount of cDNA that could be subjected to a qPCR. To overcome such limitations to some extent, cells to be evaluated should be split into multiple wells and run qPCRs in dozens of wells. It is also critical to develop a method much more sensitive than qPCR to address such limitations. Nevertheless, our method provides simple, highly-sensitive and robust method for validating, and, in turn, enhancing the safety of cell product by facilitating the exclusion of contaminants such as undifferentiated iPSC. We have previously developed a method for generating multicellular 3D miniaturized liver primordia organoids (iPSC-liver bud: iPSC-LB) from pluripotent stem cells^13,14^. In addition, we recently reported the generation of an iPSC-liver buds entirely from iPSC (all iPSC-LB)^15^, including iPSC-HE, iPSC-EC and iPSC-STM/MC. The method presented here could be applied in the validation of the safety of all-iPSC LBs in clinical settings.

## Methods

### Human iPSC and their culture

Human iPSC lines, TkDA3-4 was kindly provided by University of Tokyo (Dr. Koji Eto and Dr. Hiromitsu Nakauchi), and 1231A3, 1383D2, 1383D6, Ff-I01, and Ff-I01s04 were provided by Kyoto University (Dr. Shinya Yamanaka, Dr. Keisuke Okita, and Dr. Masato Nakagawa). All iPSC lines were maintained on Laminin 511-E8 fragment (iMatrix-511™, kindly provided by Nippi,Inc.)-coated dishes in StemFit® AK02N (Ajinomoto Co., Inc.). A detailed procedure for differentiating hepatocytes has been described previously^15^. Briefly, the cells were incubated in RPMI 1640 (Thermo Fisher Scientific) supplemented with 2% B27, 50 ng/ml Wnt-3a, and 100 ng/ml activin A for six days to derive the DE. KnockOut DMEM medium (Thermo Fisher Scientific) supplemented with 20% KnockOut serum replacement (Thermo Fisher Scientific), 1% DMSO, 0.1 mM 2-ME, 0.5% L-glutamine, and 1% NEAAs was used to derive the HE and IHs. HBM (Lonza Bioscience) supplemented with the Single Quotes^TM^ kit without EGF (HCM without EGF), 5% fetal bovine serum (FBS), dexamethasone, and OSM was used to derive MHs.

The use of human iPSC was approved by the ethical committee at Yokohama City University and the University of Tokyo.

### Detection of residual undifferentiated iPSC

To detect and quantify the residual undifferentiated cells in differentiated cells, we employed a method described by Tano *et al*.^22^. Briefly, cells were dissociated with trypsin-EDTA and seeded with StemFitAK02 medium on lamin511-E8-coated dishes in the presence of Rock inhibitor Y-27632 at a density of 8 × 10^4^ cells/cm^2^. The medium was changed every day with StemFitAK02 medium without Rock inhibitor. After cultivation for seven days, the cells were immunostained with SOX2 antibody and SOX2 + colonies were counted. One colony was considered derived from one residual undifferentiated cell.

### Immunostaining

Cells were fixed with 4% paraformaldehyde for 15 min, washed twice with phosphate-buffered saline (PBS). Cell membranes were permeabilized with 0.1% TritonX-100 in PBS (PBST) for 10 min and blocked with 5% FBS in PBST. Primary antibodies against SOX2 and TRA-1-60 were applied (Cell Signaling Technologies) followed by staining with secondary antibody.

### Microscopy and colony counting

Whole-well images after SOX2 and Tra-1-60 immunostaining were obtained using a fluorescence microscope (BZ-X710, KEYENCE, Osaka, Japan). Colonies were counted manually and described in numbers per square cm.

### Undifferentiated iPSC spike-in

Undifferentiated iPSC were spiked-in, i.e., mixed into HE cells at proportions ranging from 0.0025% to 5% as indicated in the figures.

### Human iPSC-EC, STM and NSC differentiation

Endothelial cells and mesenchymal cells were derived as described previously^15^. iPSC-NSC were derived as described previously^31^. Gene expression was evaluated using qPCR and flow cytometry. Following antibodies were used for flow cytometric analysis: PE-conjugated mouse anti-human CD140b (PDGFRB; 558821, BD Biosciences); APC-conjugated anti-human CD166 (ALCAM; 130-119-809, Miltenyi Biotec); anti- human CD105, human CD73, and human CD90 (MSC phenotyping kit; 130-095-198, Miltenyi Biotec); FITC-conjugated mouse anti-human CD31 (557508, BD Biosciences); PE-conjugated mouse anti-human CD144 (561714, BD Biosciences).

### Human iPSC- trilineage differentiation

Alternative three-germ layer derived cells were differentiated using a commercially available kit, STEMdiff Trilineage Differentiation Kit (Stem Cell Technologies). Cells were derived according to manufacturer’s instructions. Gene expression was evaluated using qPCR. Immunostaining was performed using a FOXA2 (07-633, Millipore) and a SOX17 (AF1924, R&D) for the endoderm, T (AF2085, R&D) and NCAM (362502, BioLegend) for the mesoderm, PAX6 (ab5790, abcam), and a NESTIN (MAB5326, Millipore) for the ectoderm.

### Gene expression analysis

For gene expression analysis of developing the mouse hepatocyte, published microarray data were reanalyzed (GSE46631)^13^. Published single cell RNA sequence data (GSE81252 and GSE96981) were reanalyzed using R studio (https://www.rstudio.com/)^14^. qRT-PCR analyses were performed according to standard procedures using an RNeasy Mini Kit (Qiagen) and the Universal ProbeLibrary (Roche). smFISH procedures were performed using branched DNA probes as described previously^32^.

### Droplet digital PCR (ddPCR)

The cDNA was prepared as described above. The ddPCR reaction mixtures were composed as follows: 1 × ddPCR Supermix for Probes (No dUTP) (Bio-Rad), 1 μM forward and reverse primers, and 250 nM UPL probe (Roche), and 12.5 ng of cDNA. The Droplets were generated using a QX200 droplet generator (Bio-Rad) and PCR reaction was performed according to the manufacturer’s instructions. Thermal cycling conditions were as follows: 10 min at 95 °C, 40 cycles of a thermal profile comprising 15 s at 95 °C and 60 s at 60 °C, and followed by 10 min at 98 °C and kept at 15 °C. After the PCR, the samples were analyzed using a QX200 Droplet Reader (Bio-Rad) and QuantaSoft (Bio-Rad). The fluorescence amplitude thresholds were manually determined for each gene by comparing the distribution of the signals of negative controls (distilled water). The number of copies of target per cell (estimated as 10 pg of RNA/cell^33^) was calculated as concentration (cps/20 μL)/(12.5 ng/20 μL)/1000 × 10.

### Statistics

Data are expressed as means ± SD of independent experiments. Statistical significance was assessed using the Student’s *t*-test for differences in gene expression and statistical significance of the differences in the amounts of albumin produced were assessed using the non-parametric Mann–Whitney U test. Two-tailed p values <0.05 were considered significant.

## Supplementary information

SUPPLEMENTARY INFO.
