# Supplementary material for: Robust detection of undifferentiated iPSC among differentiated cells
Source: Sci Rep. 2020 Jun 24;10:10293. doi: 10.1038/s41598-020-66845-6 (PMC7314783; doi:10.1038/s41598-020-66845-6)
Supplement: Supplementary file 1 — SUPPLEMENTARY INFO. [file 41598_2020_66845_MOESM1_ESM.pdf]

Robust detection of undifferentiated iPSC among differentiated cells

Keisuke Sekine, Syusaku Tsuzuki, Ryota Yasui, Tatsuya Kobayashi, Kazuki Ikeda, Yuki Hamada, Eriko Kanai, J. Gray Camp, Barbara Treutlein, Yasuharu Ueno, Satoshi Okamoto, Hideki Taniguchi

Supplementary Figure Legends

Figure S1

Validation of differentiated cells toward hepatic lineage. Gene expression analysis of the undifferentiated iPSC (iPSC), Definitive endoderm (DE), hepatic endoderm (HE), immature hepatocyte (IH) , and mature hepatocyte (MH).

Figure S2.

Definitive endoderm (DE), hepatic endoderm (HE), and immature hepatocyte (IH) mature hepatocytes (MHs)

Novel markers were down-regulated during differentiation in five different iPSC clones. #1-#5 represents Ff-I01, Ff-I01s04, 1231A3, 1383D2 and 1383D6, respectively.

Figure S3.

Estimated actual RNA count of the markers in one undifferentiated iPSC by droplet digital PCR.

Figure S4.

Correlation between doubling time at undifferentiated iPSC and residual undifferentiated cell number at HE (left). Correlation between passage number of undifferentiated iPSC and its doubling time (right).

Figure S5.

Marker gene expression in three germ lineages derived using different differentiation methods. a-c) represent adequate differentiation of trilineage. bar: 100um d) Marker gene expression in each lineage evaluated by qPCR. e) Actual residual undifferentiated cell number per cm2 when cells were seeded at a density of $8x10^4$ cells/cm^2.

Figure S1

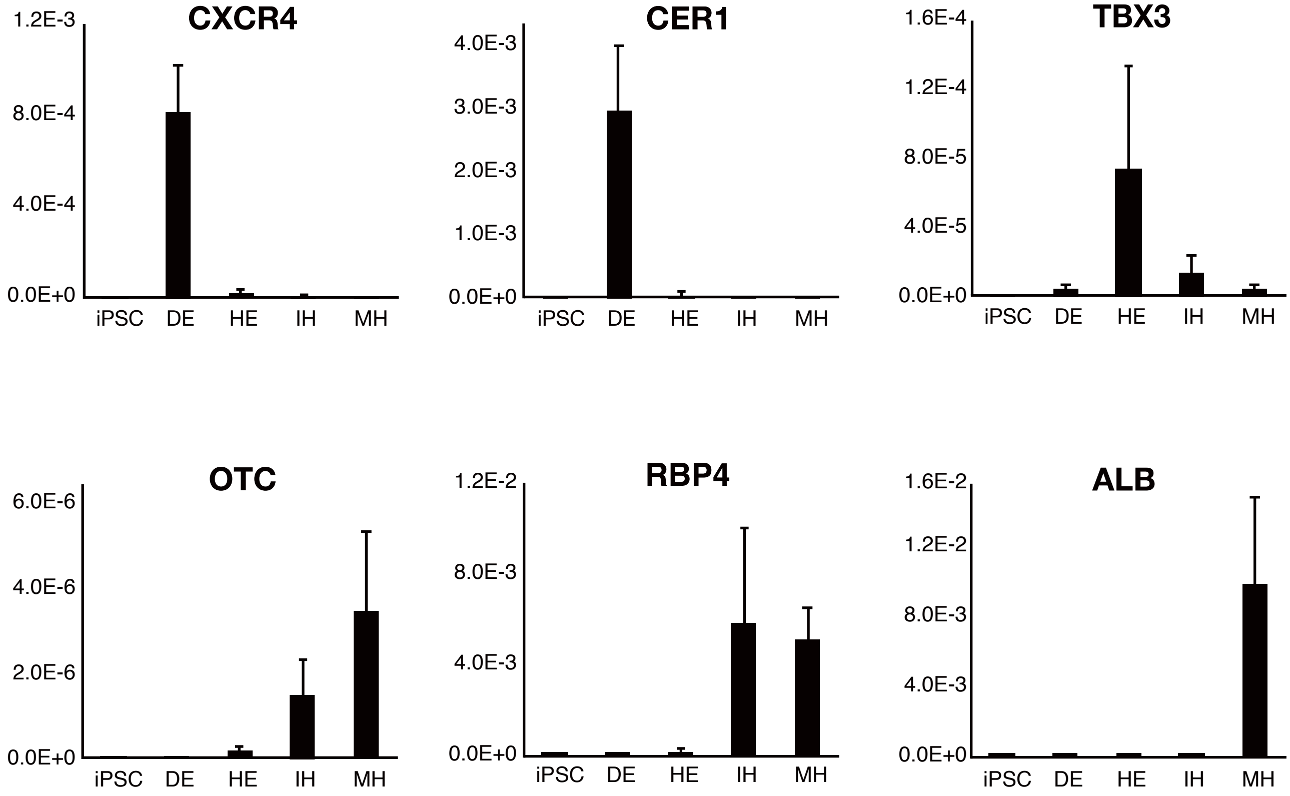
CXCR4
1.2E-3
8.0E-4
4.0E-4
0.0E+0
iPSC
DE
HE
IH
MH
CER1
4.0E-3
3.0E-3
2.0E-3
1.0E-3
0.0E+0
iPSC
DE
HE
IH
MH
TBX3
1.6E-4
1.2E-4
8.0E-5
4.0E-5
0.0E+0
iPSC
DE
HE
IH
MH
OTC
6.0E-6
4.0E-6
2.0E-6
0.0E+0
iPSC
DE
HE
IH
MH
RBP4
1.2E-2
8.0E-3
4.0E-3
0.0E+0
iPSC
DE
HE
IH
MH
ALB
1.6E-2
1.2E-2
8.0E-3
4.0E-3
0.0E+0
iPSC
DE
HE
IH
MH

Figure S2

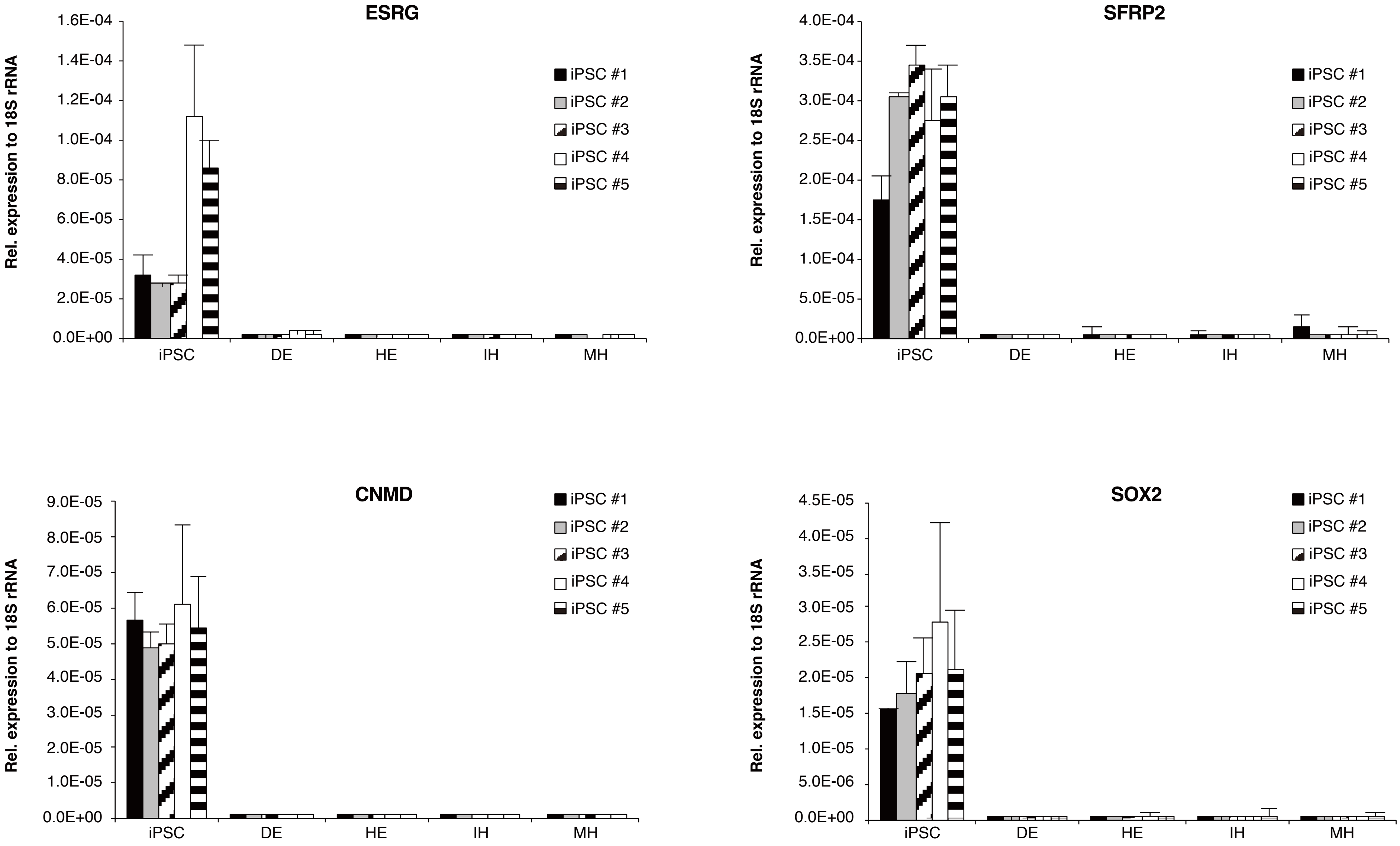
ESRG
SFRP2
CNMD
SOX2
Rel. expression to 18S rRNA
iPSC #1
iPSC #2
iPSC #3
iPSC #4
iPSC #5
iPSC
DE
HE
IH
MH
1.6E-04
1.4E-04
1.2E-04
1.0E-04
8.0E-05
6.0E-05
4.0E-05
2.0E-05
0.0E+00
4.0E-04
3.5E-04
3.0E-04
2.5E-04
2.0E-04
1.5E-04
1.0E-04
5.0E-05
9.0E-05
7.0E-05
5.0E-05
3.0E-05
1.0E-05
4.5E-05
3.5E-05
2.5E-05
2.0E-05
1.5E-05
1.0E-05
5.0E-06

Figure S3

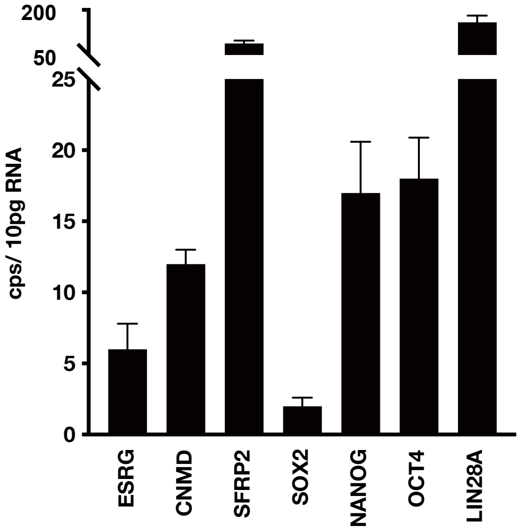

200
50
25
20
15
10
5
0
cps/ 10pg RNA
ESRG
CNMD
SFRP2
SOX2
NANOG
OCT4
LIN28A

Figure S4

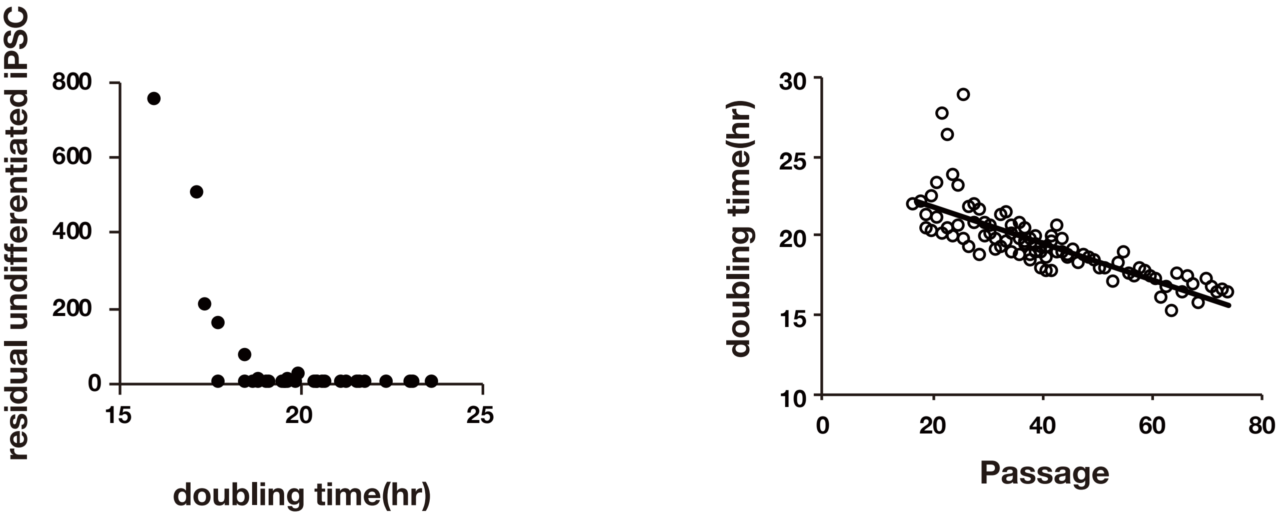

residual undifferentiated iPSC
800
600
400
200
0
15
20
25
doubling time(hr)
doubling time(hr)
30
25
20
15
10
0
20
40
60
80
Passage

Figure S5

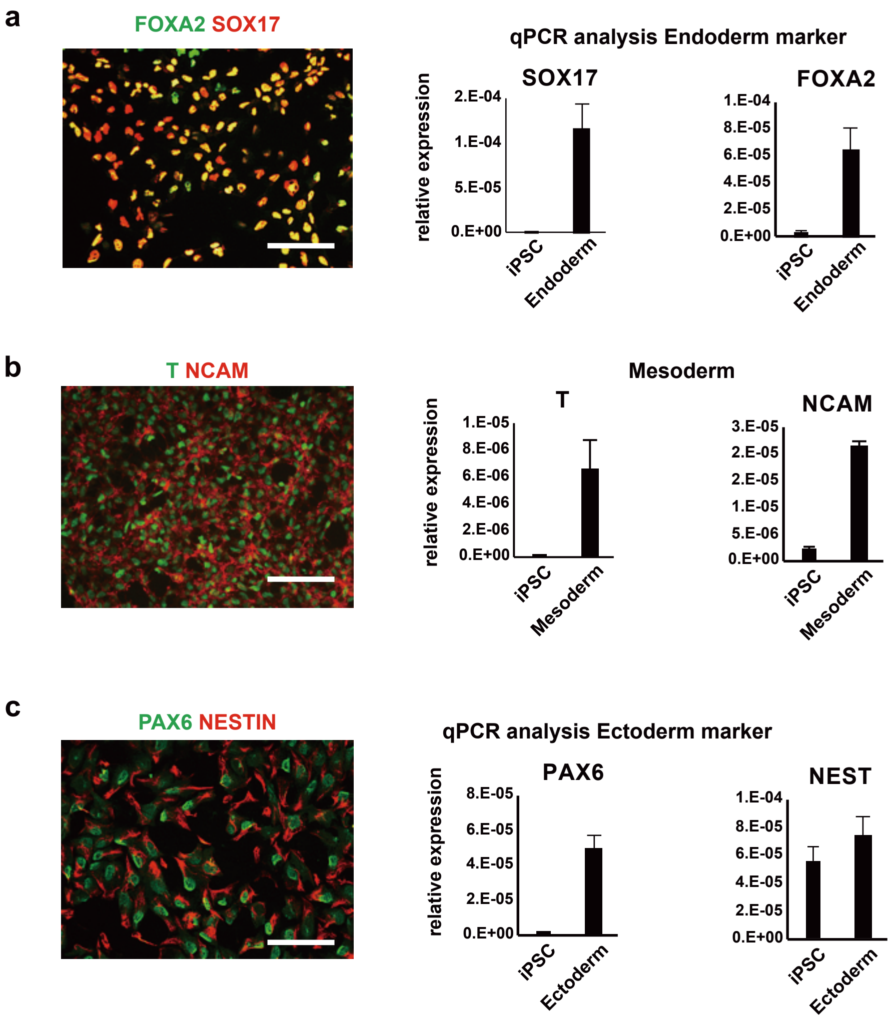

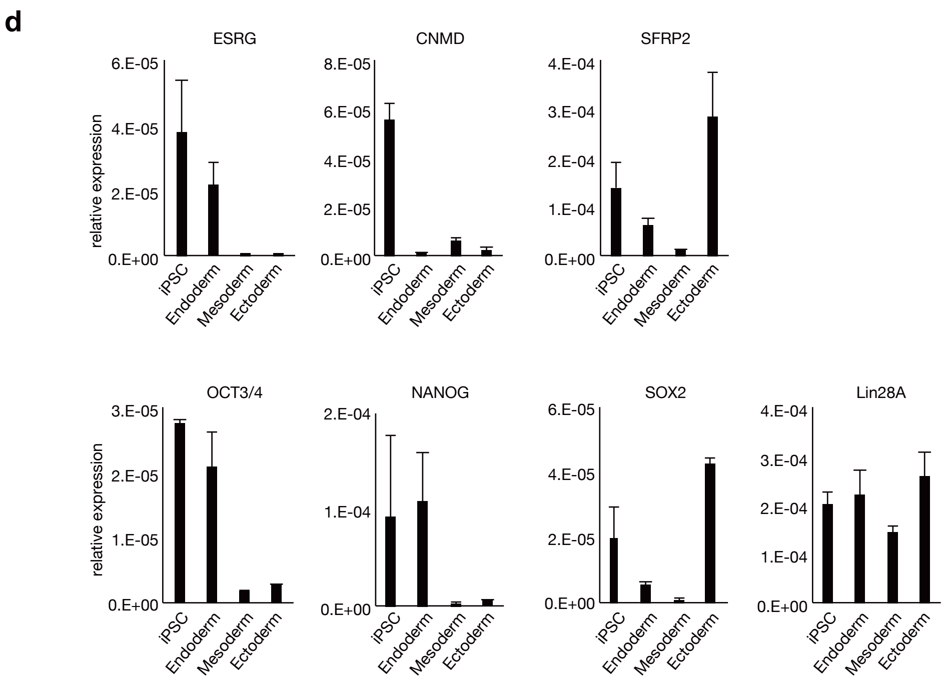

e

n=4	Colony number (colonies/cm2)
Endoderm	236.88 ± 33.41
Mesoderm	0 ± 0.00
Ectoderm	0 ± 0.00
